# Female genital tuberculosis cases with distinct clinical symptoms: Four case reports

**Published:** 2018-01

**Authors:** Gonul Aslan, Mahmut Ulger, Seda Tezcan Ulger, Huseyin Durukan, Faik Gurkan Yazici, Gurol Emekdas

**Affiliations:** 1 *Department of Medical Microbiology, Faculty of Medicine, Mersin University, Mersin, Turkey.*; 2 *Department of Pharmaceutical Microbiology, Faculty of Pharmacy, Mersin University, Mersin, Turkey.*; 3 *Department of Gynaecology Obstetrics and Reproductive Medicine, Faculty of Medicine, Mersin University, Mersin, Turkey.*; 4 *Department of Medical Microbiology, Faculty of Medicine, Biruni University, Istanbul, Turkey.*

**Keywords:** Female genital tuberculosis, Mycobacterium tuberculosis, Infertility

## Abstract

**Background:**

Tuberculosis (TB) is an infectious disease caused by Mycobacterium tuberculosis. Genital TB (GTB) is a form of extrapulmonary TB that occurs more frequently in women, in whom it classically presents in association with menstrual irregularity, pregnancy loss and short and long-term sequelae especially infertility in infected women. Patients with GTB are usually young women diagnosed during workup for infertility. GTB is rare in postmenopausal women and responsible for only approximately 1% of postmenopausal bleeding. In this study, we aimed to evaluate the laboratory, clinical and demographic characteristics of female GTB cases.

**Case:**

We presented four female GTB cases with distinct clinical symptoms. All patients have no history of TB, and no acid-fast bacilli were seen in smears prepared from the clinical materials of the patients. Histopathological examinations revealed granulomatous inflammation in all patients.

**Conclusion:**

In the light of the clinical features of these cases we aimed to emphasize that, female GTB must be taken into account in the patients with different clinical symptoms like postmenopausal bleeding, menometrorrhagia, infertility, and menstrual irregularities. We believe that these symptoms will be helpful for the diagnosis and treatment of female GTB.

## Introduction

Tuberculosis (TB) is a contagious disease caused by Mycobacterium tuberculosis (M. tuberculosis). TB remains a major global health problem, and after the human immunodeficiency virus it is the most common infectious disease causing death. According to a global TB report, there were an estimated 10.4 million new (incident) TB cases worldwide there were an estimated 1.4 million TB deaths in 2015 (1). Pulmonary TB is the most common form, but TB can affect the other parts of the body (2). Female genital TB (GTB) is an important disease and in infected women it causes morbidity, menstrual irregularity, pregnancy loss, short and long-term sequelae (2-4).

Symptoms of GTB are usually non-specific, for this the prevalence of GTB is less than expected (5). In this form of infection, fallopian tubes (95-100%), endometrium (50-60%) and ovaries are the most affected areas (6). GTB is seen approximately 5-10% in infertile females; while the incidence of GTB in the industrialized countries is about 1% this ratio is about 13% in the developing countries. This disease is generally affect the women aged between 20-40 yr (7). GTB mostly seen in reproductive age group while it could be seen in postmenopausal women as well (8). Like peritoneal carcinosis or ovarian malignancy, abdominopelvic TB has similar symptoms and the common symptoms of advanced ovarian carcinoma as pelvic pain, mass, ascites, and elevated serum CA125 (range: 0-35 IU/ml) levels are simlar to the peritoneal TB (9). 

In this study, we aimed to evaluate the laboratory, clinical and demographic characteristics of four female GTB cases with distinct clinical symptoms. Oral consent was obtained from all patients who participated in the study.

## Case report

From January 2005 to August 2012, 40 patients suspected to TB from gynaecology ward, totally 63 clinical materials [abscess (n=2), sputum (n=26), biopsy (endometrial and peritoneal) (n=6), urine (n=6), peritoneal fluid (n=18), pleural fluid (n=4), cervical discharge (n=1)] were sent to Mycobacteriology Laboratory, Department of Medical Microbiology, Faculty of Medicine, University of Mersin. After homogenization and decontamination procedure (10) smears were prepared for Ehrlich-Ziehl-Neilsen (EZN) stain. M. tuberculosis culture was performed with Lowenstein-Jensen (LJ) medium and Mycobacteria Growth Indicator Tube (MGIT). Cultures were incubated at 37oC for at least 6 weeks and inspected weekly for bacterial growth. The presence of mycobacteria in positive cultures was confirmed by EZN staining method (11).

Acid-fast bacilli (AFB) smear-positive colonies were considered as positive and conventional biochemical methods were used for further identification. The isolates were examined for their catalase activity, niacin accumulation, nitrate reduction, growth on Para-nitrobenzoic Acid Medium, microscopic and macroscopic colony morphology on LJ medium (12). Polymerase Chain Reaction (PCR)-Restriction Fragment Length Polymorphism (RFLP) assay was used for molecular identification to the species level (13). For quality control, M. tuberculosis H37Rv was used as during conventional and molecular identification. Anti-TB drug susceptibility to the first line antibiotics (Streptomycin [SM], Isoniazid [INH], Rifampicin, and Ethambutol) was performed with MGIT system according to the manufacturer’s recommendations.

Smears prepared for EZN staining were examined and no AFB was detected. Only four (10%) patients were positive by culture method (LJ and/or MGIT). With conventional biochemical methods and PCR-RFLP method, all strains were identified as Mycobacterium tuberculosis complex (MTC). According to the anti-TB drug susceptibility results, one strain was resistant to INH, one was resistant to SM and the other two strains were susceptible to the all first-line anti-TB drugs. Patient characteristics are summarized in Table I.

Case 1: A 71-yr-old woman presented with postmenopausal bleeding. Gynecological examination revealed endometrial thickening. There was no history of contact with TB patient. Histopathological examination of the endometrial biopsy specimen revealed granulomatous endometritis and endometrial polyps. After homogenization and decontamination procedure, biopsy material was stained with EZN and no AFB was seen. M. tuberculosis culture was performed with LJ medium and MGIT. Only MGIT liquid medium was positive. According to the identification with conventional biochemical methods and PCR-RFLP, the strain was identified as MTC. Anti-TB susceptibility was performed and the strain was sensitive to all first-line anti-TB drugs.

Case 2: A 31-year-old woman with primary infertility presented with a complaint of vaginal discharge and secondary amenorrhea for the past three years. Like case 1, there was no history of contact with TB patient. Endometrial biopsy was performed. Histopathological examination revealed chronic granulomatous inflammation. No AFB was seen after homogenization and decontamination procedure. In this case, both LJ and MGIT mediums were positive. The strain was identified as MTC and anti-TB susceptibility was performed. Only INH resistance was detected in this strain.

Case 3: A 22-yr-old virgin female presented with lower abdominal pain with menstrual irregularities. She took non-specific antibiotics for 15 days. Family history was negative for TB. There was a complex mass of 7 cm with solid and cystic components in the right adnexa. CA125 level was 167 mg/dL. A peritoneal biopsy was obtained via laparotomy. AFB was not seen with EZN stain. In culture, both LJ and MGIT mediums were positive. After the identification of strain as MTC, anti-TB susceptibility was performed and SM resistance was detected solely.

Case 4: A 44-yr-old gravidity 5, primiparous female with four abortions in the first trimester and with a post-neonatal death of her child presented with irregular menstrual bleeding and abdominal pain. There was no history of contact with a TB patient. Pelvic examination revealed a pelvic mass of 10 cm and abdominal fluid collection. CA125 was 1000 mg/dL, and CA19-9 was 139 mg/dL. Endometrial biopsy was reported to be granulomatous endometritis. Four liters of serohemorrhagic fluid and miliary spread of small foci of nodules in all peritoneal surfaces including bilateral tubal structures and ovaries were encountered in explorative laparotomy. 

Examination of frozen sections demonstrated necrotizing granulomatous inflammation. Samplings of the peritoneal lesions and ascites were performed for microbiological evaluation. After homogenization and decontamination procedure, no AFB was seen from peritoneal fluid. In peritoneal fluid culture, both LJ and MGIT mediums were positive. The strain was identified as MTC. Anti-TB susceptibility was performed and the strain was sensitive to all first-line anti-TB drugs.

**Table I:** Characteristics, clinical features, and laboratory results from four women with GTB

**Characteristics/Clinical features/** **Laboratory results**		**Patient 1**	**Patient 2**	**Patient 3**	**Patient 4**
Age (yr)		71	31	22	44
Prior history of tuberculosis		None	None	None	None
Symptoms and signs at presentation		Postmenopausal bleeding	Primary infertility presented with a complaint of secondary amenorrhea	Lower abdominal pain with menstrual irregularities	Pelvic mass
Clinical material		Endometrial biopsy	Endometrial biopsy	Peritoneal biopsy	Peritoneal fluid
Histopathology		Granulomatous endometritis and endometrial polyps	Chronic granulomatous inflammation	Granulomatous salpingitis	Necrotizing granulomatous inflammation
AFB		Negative	Negative	Negative	Negative
Culture					
	LJ	Negative	Positive (27^th^ day)	Positive (20^th^ day)	Positive (30^th^ day)
	MGIT	Positive (21^st^ day)	Positive (17^th^ day)	Positive (13^th^ day)	Positive (26^th^ day)
Identification (conventional biochemical methods/PCR-RFLP)		MTC	MTC	MTC	MTC
Anti-TB drug susceptibility		SM: S INH: S RIF: S EMB: S	SM: S INH: R RIF: S EMB: S	SM: R INH: S RIF: S EMB: S	SM: S INH: S RIF: S EMB: S

AFB: Acid-fast bacilli

LJ: Lowenstein-Jensen

MGIT: Mycobacteria growth indicator tube

TB: Tuberculosis

SM: Streptomycin

INH: Isoniazid

RIF: Rifampicin

EMB: Ethambutol

S: Susceptible

R: Resistant

MTC: Mycobacterium tuberculosis complex

PCR-RFLP: Polymerase chain reaction-restriction fragment length polymorphism

## Discussion

GTB cases are usually asymptomatic, for this reason clinical diagnosis of the disease is difficult. On the other hand, even if the patient is symptomatic the symptoms can be similar to other pelvic diseases symptoms (14). Among all TB cases approximately 0.5% comprise female GTB cases, and among genitourinary cases, less than 50% are GTB cases (15). Patients with GTB are usually young women diagnosed during workup for infertility (similar to case 2). Like in our case 1, in postmenopausal women this disease is seen rare and it has been reported nearly 1% of postmenopausal bleeding reason is this disease (8). Like PTB, GTB is also usually related to M. tuberculosis and female GTB is usually a secondary complication of pulmonary or extrapulmonary TB forms located other than the genital tract (14, 15).

The TB bacilli can spread from the lungs, lymph nodes and skeletal system to the genital tract and organs by the hematogenous way or directly from the gastrointestinal tract, mesenteric nodes or peritoneum (2, 15). In GTB nearly 95-100% of the patients, the fallopian tube forms the primary focus, followed by uterus and ovaries in 50-60% and 20-30% respectively (2).

Genital TB tends to be an indolent infection and the commonly reported symptoms were infertility (44%) and pelvic pain (25%), followed by vaginal bleeding (18%), amenorrhea (5%), vaginal discharge (4%) and postmenopausal bleeding (2%) (8, 15). Like these symptoms, in our case 3, the patient had lower abdominal pain with menstrual irregularities. Less often symptoms as abdominal mass, ascites, Tubo-ovarian abscess and vague abdominal distention were reported (8). In our case 4, a patient with no prior TB contact had four abortions in the first trimester after a normal course of pregnancy and birth. 

The diagnosis of GTB is a clinical challenge and for this, the diagnosis of this disease is made by a combination of tests (2). In order to make an accurate diagnosis of GTB, clinicians requires some additional data derived from abdominopelvic ultrasonography, chest radiography, tuberculin skin test, hysterosalpingography for beading, AFB staining, histopathological evaluation and specific cultures from intra-operative specimens obtained from diagnostic laparoscopy (2, 14, 15).

## Conclusion

In conclusion, female GTB must be taken into account in the patients with different clinical symptoms like postmenopausal bleeding, menometrorrhagia, infertility and menstrual irregularities. We believe that these clinical symptoms will be helpful for the diagnosis and treatment of female GTB.
